# News and Events of the Week

**Published:** 1910-05-28

**Authors:** 


### News and Events of the Week.

### The Death of King Edward VII.

The *Court Circular* of last Tuesday contained the following note : Queen Alexandra is very grieved that a report has been printed and circulated by some society that the death of the late King was caused by a vaccine treatment he had received to prevent his contracting influenza or pneumonia before his Majesty's journey to Biarritz. Queen Alexandra wishes it to be known that before the late King left England he had never felt better in health and spirits than after this treatment, for it had kept him in excellent health, to his Majesty's entire satisfaction, for no less than fifteen months. Queen Alexandra wishes it stated that his Majesty's attack at Biarritz was in no way related to his previous course of treatment.

The following resolution was passed at the annual meeting of the Pharmaceutical Society on Wednesday of last week and sent to King George V. : "That the members of the Pharmaceutical Society of Great Britain in annual general meeting assembled, pursuant to Royal Charter of Incorporation, desire most humbly and sincerely to express the profound sorrow and sense of loss felt by all classes of the pharmaceutical community at the death of his late Majesty King Edward VII., and to tender respectful and heartfelt sympathy to your Majesty and the members of the Royal Family, together with unfeigned assurances of loyalty and duty."

The Pure Food Exhibition at the Royal Horticultural Hall, Westminster, was opened, without public ceremony, last Monday.

Mr. Lawrence Jones has resigned the post of assistant-surgeon to S. George's Hospital, which he had held since 1905. Over a year Mr. Jones had to give up work on account of ill-health, and although his general condition has improved very markedly since he left the hospital, his advisers do not think it right for him to resume active work in London.

Sir Donald MacAlister, presiding at the annual meeting of the General Medical Council, referred sympathetically to the death of King Edward, and the Council resolved to tender to his Majesty an address of sympathy and devotion.

In a recent letter to the *Times*, Lord Walsingham states that so long ago as 1892 his attention was attracted to the condition of the road dust in the avenue of lanes to the west of Cannes, and he attributed a serious attack of pneumonia following severe bronchial and catarrhal irritation in a member of his family to this cause. The microscope applied to this dust, which was flying in clouds on a high wind, showed a plentiful admixture of minute spicules formed by the breaking up of the fruit balls of plane trees; and since that time innumerable instances have occurred in various places to confirm his impression then formed; indeed, it may be stated broadly that wherever plane trees are abundant, colds, coughs, inflamed eyelids, throat troubles, and general irritation of the mucous membrane are more prevalent than where such trees do not exist. In "Trees of Great Britain" it is stated on good authority that "Plane trees are prohibited by law from being planted near or around schools in Alsace-Lorraine," and in the *Gardener's Chronicle*, iii. 370 (1888), it is shown that Galen and Dioscorides knew of the irritating qualities of the plane in ancient Greece. It had been supposed that this irritation was caused by the minute hairs shed by the pubescent leaves when first expanding in spring; but a case of severe bronchial irritation which lately occurred at Acqui in North Italy, probably due to a drive in the celebrated avenue of planes on a windy day, could not be thus attributed since the leaves had not yet appeared. Dr. Henry, reader in forestry at Cambridge University, exposed, during April, glass slides smeared with glycerine at Kew and in Berkeley Square, and found that these exhibited numerous particles of the broken fruit spicules.

Dr. Thomas James Higgins, M.D., of Bridge Street House, Louth, Lincolnshire, hon. surgeon to the Louth Hospital, surgeon to the Great Northern Railway, medical officer and public vaccinator for the Welton-le-Wold district, who died on March 18 at the age of 71, left estate valued at £18,749 gross and £11,963 net.

The Coroner for South Bucks held an inquiry on May 13 into the death of Miss Lyndall Rice, aged eighteen, medical student at the London School of Tropical Medicine, Hunter Street. One night last week her parents found her sitting in a chair apparently asleep, but really dead. On a table was a bottle that had contained an iron tonic which Miss Spencer, a lady chemist, had given her for anæmia. There was a small quantity of liquid in it, which Miss Spencer declared was not the tonic which she had given Miss Rice, but prussic acid. Dr. Thompson Bell said he was not prepared to swear that it was that drug. Miss Spencer said Miss Rice had previously asked to be supplied with chloroform to kill a cat for dissecting purposes, but she refused, as the girl was under age. Dr. Bell said he had made a post-mortem examination, but could come to no conclusion as to the cause of death. The inquiry at length was adjourned for a fortnight that the county analyst might analyse the internal organs.

All those who look forward with pleasure to the commencement of the daily sea trips down the River Thames, will be pleased to hear that the new Palace steamer *Royal Sovereign* commenced sailings to Southend, Margate, and Ramsgate on May 14, and her sister ship, the *Koh-i-noor*, will commence her regular sailings to Deal and Dover on June 19. Both the steamers have been thoroughly overhauled during the winter months, and have passed all the requirements of the Board of Trade for their passenger certificates. That popular Saturday afternoon trip, the "Husbands' Boat," will commence on June 18, and continue throughout the season. The circular bookings with the South Eastern and Chatham Railway, down by boat and home by rail, which have proved so popular in past years, have again been arranged for. The catering, so essential to the perfect enjoyment of a holiday, is controlled by the company, wholesome food and drink being supplied at strictly moderate charges.

The annual Whitsun conference of the Hearts of Oak Benefit Society was concluded last week. Mr. J. N. Lee presided, there being again a large attendance of delegates. A letter was read from Dr. R. J. Collie, chief medical officer, resigning his appointment, which the Executive Council accepted. The delegates instructed the Council to appoint a successor. A report was submitted upon tuberculosis sanatoria by Mr. T. Stops, chairman of that committee. He observed that a number of delegates of various friendly societies attended on the Continent and inspected various places of open-air treatment for that malady. Since their visit, the Benenden Sanatorium had been established, and sixty-three beds were now maintained by the friendly societies of this country, and the Oddfellows were also increasing their support. He urged that it was much needed that the medical gentlemen who examined or sent members to Benenden should exercise extreme care in recommending patients to the institution. Sometimes members were sent who should have gone to a home for incurables. In his opinion it was one of those matters that was too big for the friendly societies to deal with, and pressure should be brought to bear upon the Government to deal effectively with the question.

The seventh ordinary meeting of the Royal Statistical Society was held at 5 p.m. on Tuesday, May 24, 1910, at the rooms of the Royal Society of Arts, John Street, Adelphi, W.C., when Mr. G. H. Wood read a paper on "Statistics of Wages in the Nineteenth Century, Part XV. The Cotton Industry Section. Changes in the Average Wage of all Employed, with some Account of the Forces Operating to Accelerate or Retard the Progress of the Industry." Tea was served at 4.30 p.m. at 9 Adelphi Terrace.

The annual report for the year 1909 of the Medical Defence Union, which has just been issued, shows that nothing occurred during that year to mar the success of the Union; in fact, "the Union," to quote from the legal statement made by the solicitor, "has during 1909 preserved an unblemished record, and not one drop of blood, much less a pound of flesh, has been extracted from any member whose case has been undertaken and defended within the past year." The Union has now been established for nearly a quarter of a century, and possesses a membership equivalent to about one-fourth of the registered practitioners practising within the United Kingdom. Of the 158 legal cases falling within the last year's work, 137 were actually completed, leaving in hand at the end of the year 21 cases to be finally dealt with or adjusted. It is interesting to take a glance at the class of cases which the Union found it necessary to take up; 46 of these related to the suppression of libels and slanders upon members, 40 involved the defence of members in actions for alleged negligence or want of care and skill in which pecuniary damages were claimed, 10 were directed to the suppression of unqualified practice and the prosecution of unregistered practitioners, the remaining 62 concerning matters personal to the interests of members as distinguished from those embraced under the heads given above. Of these last-mentioned 62 cases no less than 28 had reference to the adjustment of partnership questions and disputes arising between members of the profession, 12 to matters associated with appointments held by members in connection with which their rights were sought to be invaded or otherwise affected to their prejudice, the remaining 22 cases embracing questions arising under the Diseases Notification Acts, the Notification of Births Act, claims of members for fees for giving evidence in their professional capacity and other matters touching them in their professional life. Good service has been rendered in the interests of the profession by the prosecution of unregistered persons, and various convictions have been secured which should tend to operate in aid of the suppression of unqualified practice. The report of the Finance Committee shows that the law costs in cases placed in the hands of the solicitors in the interests of the members involved in litigation have been less than in the previous year by £695 11s. 5d. The number of members on the Register of the Union is 7,982. The number of new members last year was 425; the amount of the Guarantee Fund is £10,881 17s.

In order to protest against the form in which the examination known as the *concours d'agrégation* is being conducted under new regulations, writes the Paris correspondent of the *Times*, the medical students resumed their demonstrations of last year at the Sorbonne on the 22nd inst. In the great hall of the University in which the *viva voce* examination of candidates for medical degrees was to have begun on that day, several hundred students rendered impossible the work of the examiners by pelting them with rotten eggs and other refuse to the accompaniment of cat-calls and hisses. After a four hours' bombardment, during the course of which the Dean of the Faculty, Professor Landouzy, and several of his colleagues were repeatedly struck by projectiles and were ultimately compelled to remove their saturated gowns, the "examination" was postponed until next day. The din was so deafening that all announcements had to be written on a blackboard.

His Majesty the King has been graciously pleased to award the Edward Medal of the Second Class to Mr. Evan Owen, Mr. Edmund Davies, and Mr. William Wagner Turner, M.B., under the following circumstances. On October 29, 1909, an explosion of coal-dust occurred at the Darran Colliery, Deri, in the Cardiff district, by which 27 persons lost their lives, five succumbing during the rescue operations. Mr. Evan Owen, the under-manager of the colliery, was one of the first to enter the mine after the explosion. In the course of the day he accompanied several of the rescue parties to the furthest points it was possible to reach, and several times he was partially overcome by the effects of the poisonous gases. He persisted in his efforts until late in the day, when he was persuaded to go home. Mr. Edmund Davies, the day fireman employed at the colliery, was also among the first to enter the mine and made determined and continued attempts to succour the unfortunate men who had been affected by the explosion. Like Mr. Owen, he was at times seriously affected by the noxious air, and only desisted when his services were no longer of any avail. Dr. Turner, who was the first medical man to reach the mine, displayed great courage by promptly going down the ladders in the upcast and pumping-shaft—an awkward descent to anyone unacquainted with mining work. He rendered all the assistance he could, and nearly paid for his bravery with his life, as he was severely affected by afterdamp.

Henry Werner was last week summoned before Mr. Hedderwick for wilfully and falsely pretending to be a registered medical man. Mr. Bodkin, instructed by the Medical Defence Union, appeared in support of the summons, and said that the proceedings were taken under the Medical Act of 1858, and arose out of an inquest on the body of Ernest Reich, who at the Marble Arch Hotel early in March took poison. The defendant was called in as a medical man, and he treated Reich for the poison. After some days Reich was removed to the German Hospital at Dalston, where he died. At the inquest the defendant, who was summoned to give medical evidence, gave the name of Henry Werner, and, in reply to the Coroner's questions, said that he was a registered medical practitioner. At the end of the inquest he signed the expense sheet and received a fee of 21s. as a medical witness. The case was reported in the newspapers and was noticed by Dr. Bateman, general secretary of the Medical Defence Union, and these proceedings were initiated. It was thought desirable, however, that a visit should be paid to the defendant, and as a consequence an officer of the Union visited the house at Old Quebec Street, asked for Dr. Werner, and saw the defendant, who prescribed for him and charged a fee of 5s. It could also be shown that the defendant advertised in the *Pelican* as "Dr. Werner, the well-known French specialist." The defendant pleaded "Guilty." He had had considerable medical experience on the Continent, and thought that he was entitled to practise as a medical man. Mr. Hedderwick said he regarded this as a serious case. The defendant would have to pay the full penalty of £20, with £5 5s. costs, and in default of distress suffer one month's imprisonment.

